# *Culicoides* biting midges involved in transmission of haemoproteids

**DOI:** 10.1186/s13071-020-04516-1

**Published:** 2021-01-07

**Authors:** Rita Žiegytė, Elena Platonova, Egidijus Kinderis, Andrey Mukhin, Vaidas Palinauskas, Rasa Bernotienė

**Affiliations:** 1 Nature Research Centre, Akademijos 2, Vilnius 21, 09412 Vilnius, Lithuania; 2 Biological Station Rybachy of the Zoological Institute, Russian Academy of Sciences, Rybachy, 238535 Kaliningrad Region Russia

**Keywords:** *Culicoides kibunensis*, *Haemoproteus*, Biting midges, Vector, Sporozoites, Sampling methods

## Abstract

**Background:**

*Culicoides* biting midges (Diptera, Ceratopogonidae) are known vectors of avian *Haemoproteus* parasites. These parasites cause diseases, pathology and even mortality in birds. The diversity of biting midges in Europe is great, but only four *Culicoides* species are known to be vectors of avian *Haemoproteus* parasites. In general, our knowledge about the role of the particular *Culicoides* species in the transmission of *Haemoproteus* parasites remains insufficient. Information gaps hinder a better understanding of parasite biology and the epizootiology of parasite-caused diseases. The aim of this study was to determine new *Culicoides* species involved in the transmission of *Haemoproteus* parasites.

**Methods:**

Biting midges were collected using a UV trap as well as sticky traps installed in bird nest boxes. Individual parous females were diagnosed for the presence of haemoproteids using both PCR-based and microscopic methods.

**Results:**

We collected and dissected 232 parous *Culicoides* females from 9 species using a UV trap and 293 females from 11 species from bird nest boxes. *Culicoides obsoletus* was the dominant species collected using a UV trap, and *Culicoides kibunensis* dominated among midges collected in nest boxes. PCR-based screening showed that 5.2% of parous biting midges collected using a UV trap and 4.4% of midges collected from nest boxes were infected with avian haemosporidian parasites. Haemoproteid DNA was detected in *C. kibunensis*, *Culicoides pictipennis*, *Culicoides punctatus*, *Culicoides segnis* and *Culicoides impunctatus* females. The sporozoites of *Haemoproteus minutus* (genetic lineages hTURDUS2 and hTUPHI01) were detected in the salivary glands of two *C. kibunensis* females using microscopy, and this finding was confirmed by PCR.

**Conclusions:**

*Culicoides kibunensis* was detected as a new natural vector of *Haemoproteus minutus* (hTURDUS2 and hTUPHI01). Haemoproteid DNA was detected in females from five *Culicoides* species. This study contributes to the epizootiology of avian *Haemoproteus* infections by specifying *Culicoides* species as vectors and species that are likely to be responsible for the transmission of haemoproteids in Europe.
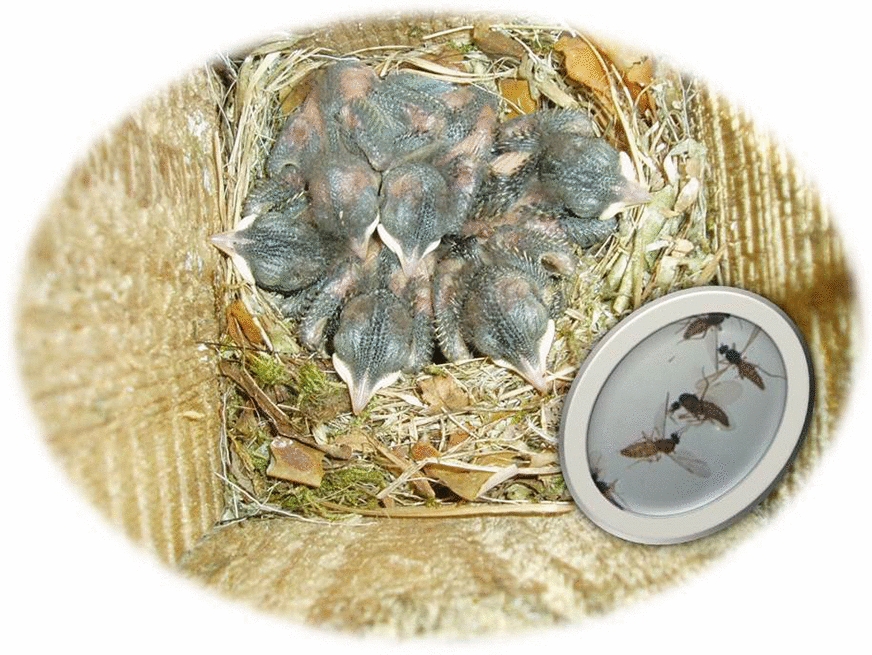

## Background

Biting midges (Ceratopogonidae: *Culicoides*) play an important role in the transmission of viruses, bacteria, parasitic protozoa and nematodes [1–3]. They are vectors of the *Haemoproteus* (Haemosporida) parasites, which can cause diseases and even lethal pathology in non-adapted birds [1–5]. At present, 1368 *Culicoides* species are known worldwide [6], but only 13 of them have been proved to support complete sporogony of avian *Haemoproteus* parasites [7, 8], though about 150 species of *Haemoproteus* have been detected in birds [9]. *Culicoides impunctatus* is one of the most abundant *Culicoides* species in North Europe [10, 11]; therefore, exhaustive experimental studies on the sporogony of haemoproteids have been performed with wild *C. impunctatus* showing that these are vectors of 12 different *Haemoproteus* species [2, 12]. Experimental studies have also been performed with *Culicoides nubeculosus*, the only Palearctic *Culicoides* species maintained in laboratories [13, 14], which have proved that *C. nubeculosus* is a vector of eight *Haemoproteus* species [7]. Recently, Bernotienė et al. [8] detected *Haemoproteus pallidus* sporozoites in wild-caught *Culicoides kibunensis* using both microscopy and PCR-based methods. Atkinson et al. [3] described the sporogonic development of haemoproteids to the infective stage (sporozoites) in *Culicoides downesi*, *Culicoides bottimeri*, *Culicoides sphagnumensis*, *Culicoides edeni*, *Culicoides hinmani*, *Culicoides arboricola*, *Culicoides haematopotus*, *Culicoides knowltoni*, *Culicoides stilobezziodes* and *Culicoides crepuscularis* biting midges. However, most of these biting midge species, except *C. sphagnumensis*, are found in North America but not Europe. Several recent studies have reported nine other *Culicoides* species as possible *Haemoproteus* vectors, but the authors used only molecular diagnosis of the parasite DNA in wild-caught insects [15–20]. Such PCR-based studies are helpful in determining ornithophagic midges but the presence of parasite DNA alone indicates only a possible vector, because PCR-based diagnostics cannot distinguish between sporozoites infective to vertebrate hosts and non-infective sporogonic stages [21]. The paucity of knowledge of the composition of *Culicoides* species involved in transmission of *Haemoproteus* parasites in the wild hinders the understanding of patterns of epizootiology [2, 3, 22].

The aim of this study was to identify biting midges obtained from a UV trap and bird nest boxes and to determine new species of *Culicoides* that could take part in the transmission of *Haemoproteus* parasites in the wild. Birds, the intermediate hosts of *Haemoproteus* parasites, are the most vulnerable to bites of *Culicoides* midges during the nesting and nestling care period [2]. This short and vulnerable period for the host was targeted in our study. First, we collected midges using a UV trap as described by Bernotienė et al. [8] and from bird nest boxes as described by Tomas et al. [23] and then sorted and identified parous females. Second, we dissected parous *Culicoides* females individually and prepared thin slides of their thorax content as salivary glands of biting midges are located in the thoraxes. Third, we applied PCR-based analysis for each collected insect to determine whether the insect was infected with *Haemoproteus* parasites. Finally, we used microscopy to examine thorax preparations of PCR-positive individuals to detect the presence of haemoproteid sporozoites. Detection of both sporozoites (the presence of infective parasite stage) and parasite DNA (molecular identification of the parasite) in the same insect allowed us to indicate natural vectors of pathogens. Detection of solely haemosporidian parasite DNA allowed indicating potential vectors of avian *Haemoproteus* parasites and required further confirmation of vector status.

## Materials and methods

### Study site and collection of biting midges

Biting midges were collected using one Onderstepoort 220 V UV trap in Verkiai Regional Park (VRP) (54°45′ N, 25°17′ E), Vilnius, Lithuania, in May–July 2018. Insects were trapped at night at least once a week. The UV trap was turned on 1–2 h before sunset and was turned off 2–3 h after the sunrise. Insects were collected in a water container supplemented with a drop of liquid soap.

**Fig. 1:**
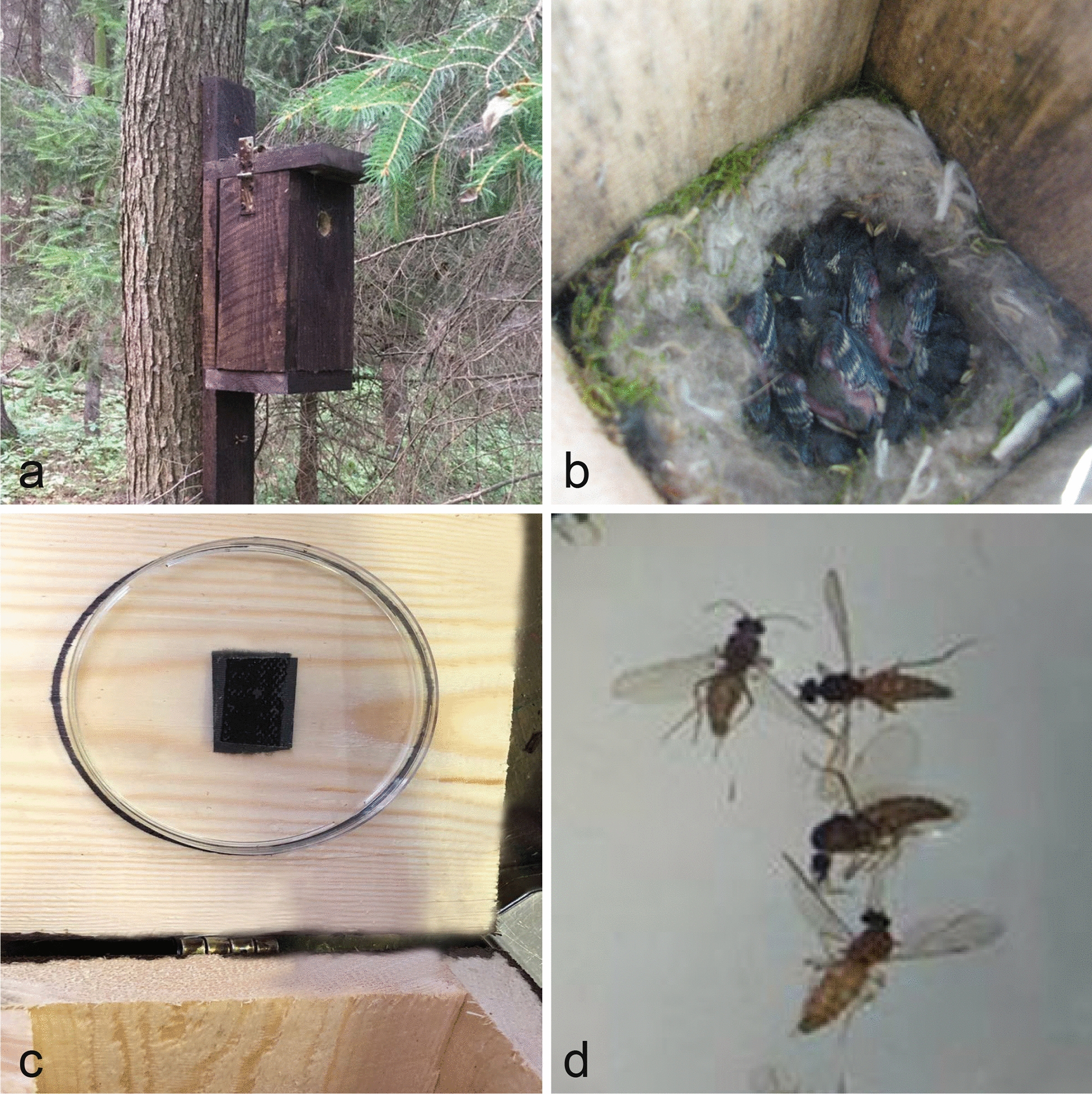
The collecting method for biting midges from bird nest boxes. Nest box attached to the tree (**a**); nestlings in the nest box (**b**); petri dish moistened with baby oil and fixed upside down using double-sided sticky tape inside the roofs of nest boxes (**c**); petri dish with sticking *Culicoides* spp. insects (**d**).

Biting midges were also collected from bird nest boxes according to the methodology described by Tomas et al. [23] in the Neris Regional Park (NRP), (54°50′ N 24°58′), Lithuania (May–June 2017), and at the Biological Station Rybachy (BSR) of the Zoological Institute of the Russian Academy of Sciences on the Curonian Spit located in the Baltic Sea (55°15′ N, 20°86′ E) in May–July 2012, 2018 and 2019. Nest boxes were attached to a tree at heights of up to 2 m (Fig. 1a). Petri dishes moistened with baby oil were temporarily fixed upside-down using double-sided sticky tape on the inside of the roofs of nest boxes (Fig. 1c). Insects flying inside the nest boxes stuck to the petri dishes and thus could be collected (Fig. 1d). Petri dishes were left overnight, as *Culicoides* biting midges are active at dusk [11], and were removed the next day. Petri dishes were replaced several times per week, in total 4−16 times per season (8.2 ± 4.3 on average). Seventy-three nest boxes with nesting adults (Fig. 1b) or hatched nestlings of *Parus major* and *Ficedula hypoleuca* were monitored: 11 in NRP and 62 in BSR (14, 28 and 20 in 2012, 2018 and 2019, respectively). Mann-Whitney *U*-tests were used to compare the number of *Culicoides* collected in nest boxes.

### Insect identification, preparation of slides and microscopic examination

Collected insects were taken to the laboratory and identified using their wing coloration and other morphological features [24–26]. Parous females were detected according to the presence of the readily visible burgundy pigmentation in the subcutaneous cells of the abdomen, indicating a digested blood meal prior to capture [27]. Heads and wings of females were removed to prepare mounted speciments in Euparal for the identification of *Culicoides* species [26].

Parous females were dissected for preparation of the thorax content as described by [2, 28]; salivary glands of biting midges are located in the front part of the thorax, and therefore the infective stage of haemoproteids (sporozoites) can be found in the salivary glands of infected biting midges [2]. Briefly, insects were placed in a small drop of 0.9% normal saline, and thorax contents were removed by light pressure on the front part of the thorax using needles. Extracted salivary glands were ground and mixed with a drop of saline. Preparations were air-dried, fixed with absolute methanol and stained with 4% Giemsa [2]. The remnants of each carcass were retained for PCR to detect the DNA of haemosporidian parasites (as described below) [29–31]. To eliminate contamination of samples, we used a new dissecting needle for each midge. All material was studied under a binocular stereoscopic microscope (Olympus SZ × 10 and Olympus B × 43 microscope, Tokyo, Japan).

### Polymerase chain reaction and sequencing

Total DNA was extracted from remnants of each individual parous midge using the ammonium acetate DNA extraction method [32].

For the detection of haemoproteids, a fragment of parasite mitochondrial DNA cytochrome *b* (*cyt b*) gene was amplified using a nested PCR protocol with outer primers HaemNFI and HaemNR3 and inner primers HaemF and HaemR2 [30, 31, 33, 34]. Primers HaemNFI/HaemNR3 amplify *cyt b* gene fragments of haemosporidians belonging to *Haemoproteus, Plasmodium* and *Leucocytozoon*, and primers HaemF/HaemR2 are specific to *Haemoproteus* and *Plasmodium* spp. To avoid false positives, we used (i) a negative control (H_2_O instead of target DNA) and (ii) a check with another set of primers: all samples positive for *Haemoproteus* spp. were double-checked using multiplex PCR primers [35], which amplify the DNA fragments between the 5′ end of *cyt b* and a non-coding region of mtDNA, which is outside of the HaemF/HaemR2 fragment.

To confirm the identification of all PCR positive for haemospordian parasites *Culicoides*, the standard mitochondrial DNA cytochrome *c* oxidase subunit 1 (*cox*1) primers LCO1490 and HCO2198 were used [29]. Morphological identification was consistent with PCR-based identification of biting midges; obtained sequences matched corresponding sequences from the GenBank 99–100%.

DNA fragments of all PCR samples were visualized on 2% agarose gel using MidoriGreen dye (NIPPON Genetics Europe, Germany). All positive samples were sequenced using both forward and reverse primers. Sequences were edited and aligned using BioEdit software [36]. Genetic lineages of parasites were identified using the ‘Basic Local Alignment Search Tool’ (megablast algorithm) (NCBI BLAST, 2019 https://blast.ncbi.nlm.nih.gov/Blast.cgi), and their identification was double checked using the MalAvi database BLAST function (http://mbio-serv2.mbioekol.lu.se/Malavi).

## Results

### *Haemoproteus* parasites in midges collected using a UV trap

In all, 232 parous *Culicoides* females were collected. We morphologically identified eight *Culicoides* species and one species complex, Obsoletus Complex, which contains several *Culicoides* species with very similar female adult morphology. *Culicoides* females belonging to Obsoletus Complex were the most abundant, forming up to 55% of all collected parous females (Table 1). Three parous midges were collected in May, 67 in July and 162 in June. We detected 12 *Culicoides* females (5.2%) PCR positive for the presence of *Haemoproteus* parasites. All these females were collected between the 19 and 28 June.

**Table 1.** *Haemoproteus* and *Plasmodium* parasites detected in collected biting midges using a UV trap in Verkiai Regional Park

Species of biting midge	No. of investigated females	No. of PCR positive insects	Parasite species and genetic lineages detected
*Culicoides festivipennis*	6	0	–
*C. impunctatus*	20	1	*Haemoproteus tartakovskyi* (hSISKIN1)
*C. kibunensis*	11	6	*Haemoproteus minutus* (hTURDUS2, hTUPHI01), *H. tartakovskyi* (hSISKIN1), *Plasmodium vaughani* (pSYAT05)
Obsoletus Complex	128	0	–
*C. pictipennis*	21	1	*Plasmodium circumflexum* (pTURDUS1)
*C. reconditus*	14	0	–
*C. segnis*	24	4	*Haemoproteus tartakovskyi* (hSISKIN1), *H. parabelopolskyi* (hSYAT01), *Haemoproteus pallidus* (hCOLL2), *Haemoproteus* sp. (hTURDUS3)
*C. fagineus*	4	0	–
*C. punctatus*	4	0	–
In total	232	12	

Six genetic lineages (haplotypes of the mitochondrial *cytb* gene) of *Haemoproteus* parasites were detected using PCR in three *Culicoides* species (Table 1): *C. kibunensis* (5 females), *C. segnis* (4 females) and *C. impunctatus* (1 female). One *C. kibunensis* and one *C. pictipennis* contained DNA of *Plasmodium* parasites (Table 1).

**Fig. 2:**
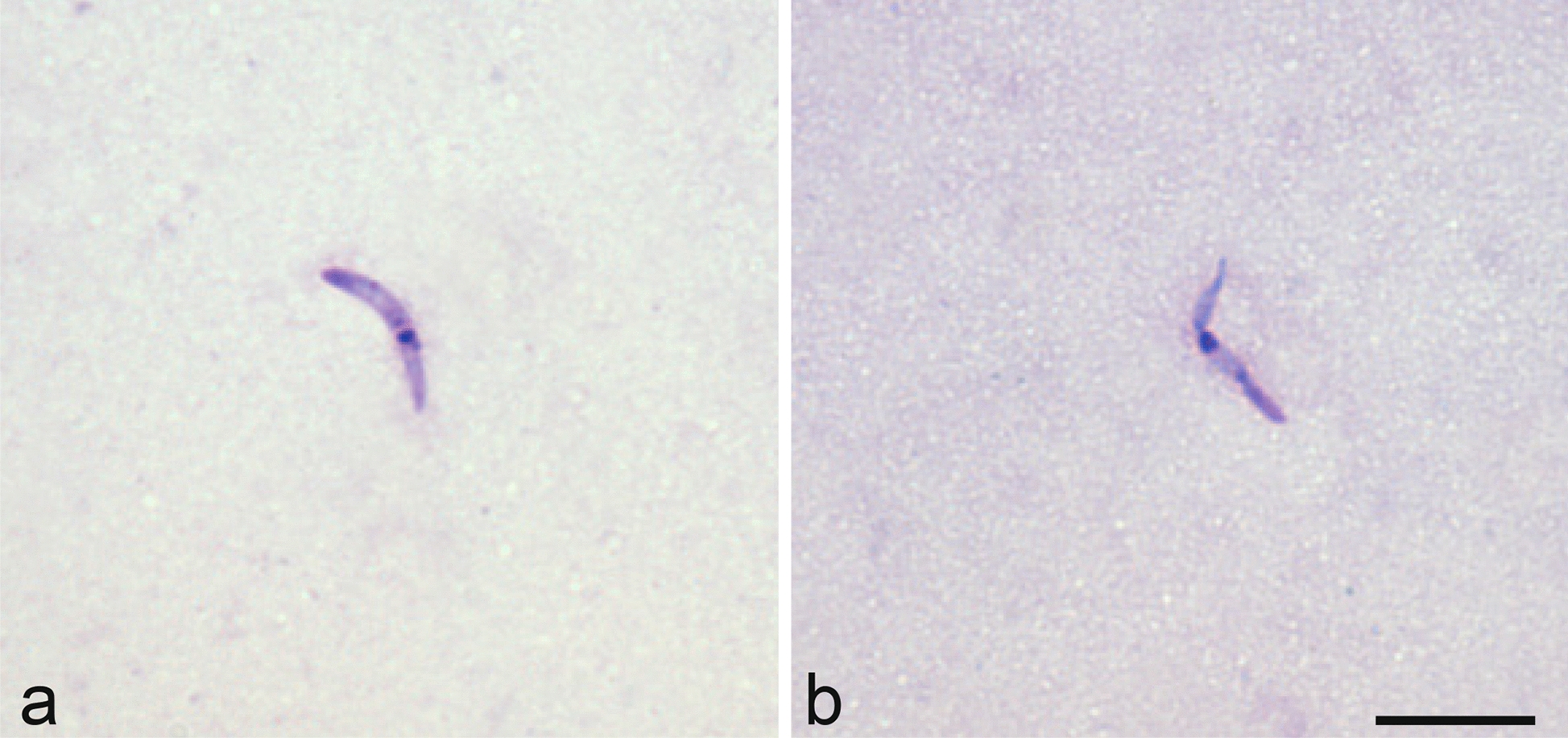
Sporozoites of *Haemoproteus minutus* in thorax preparation of *Culicoides kibunensis* (**a**, **b**). Scale bar: 10 μm

Microscopic examination of salivary gland preparations of PCR-positive *Culicoides* revealed the presence of haemoproteid sporozoites in two *C. kibunensis* biting midges (Fig. 2). Parasites identified in these two *Culicoides* were *Haemoproteus minutus* (genetic lineages hTURDUS2 and hTUPHI01).

### *Haemoproteus* parasites in midges collected from bird nest boxes

In all, 293 parous *Culicoides* females were collected from bird nest boxes with bird nestlings: 127 in the NRP and 166 in BSR (Table 2).

**Table 2.** Abundance of collected *Culicoides* biting midges and the presence of *Haemoproteus* and *Plasmodium* parasites in insects obtained from nest boxes at the Neris Regional Park and at the Biological Station Rybachy

Species of biting midge	Neris Regional Park		Biological Station Rybachy					
	2017		2012		2018		2019	
	N*	P* (genetic lineage)	N*	P* (genetic lineage)	N*	P* (genetic lineage)	N*	P* (genetic lineage)
*Culicoides festivipennis*	19						4	
*C. impunctatus*	1				1		5	
*C. kibunensis*	66	2 (pSW2, pSGS1)	11	1 (hRB1)	17		7	
Obsoletus Complex	12		3				1	
*C. pictipennis*	7		8		59	5 (hTUPHI01)	3	
*C. reconditus*	1		14	1 (pSGS1)	2			
*C. segnis*	2	1 (hCWT4)	24	2 (hSISKIN1, hTURDUS2)			2	
*C. subfascipennis*	2							
*C. sphagnumensis*			2					
*C. punctatus*			1	1 (hTURDUS2)				
*C. pallidicornis*			1					
*Culicoides* sp.	17						1	
Total	127	3	64	5	79	5	23	0

N* number of collected biting midges

P* number of biting midges positive for *Haemoproteus* and *Plasmodium* parasites

Ten *Culicoides* species and biting midges belonging to Obsoletus Complex were determined. *Culicoides kibunensis* was the most abundant species in NRP (52% of all sampled midges), while *Culicoides segnis* and *C. pictipennis* dominated in BSR (37.5% and 74.7% of all sampled midges in 2012 and 2018, respectively) (Table 2). The abundance of biting midges was very low in 2019 even though the same locality and sampling methods were used and *Culicoides* were collected at the same time as in 2012 and 2018 (Table 2). *Culicoides kibunensis* was the most abundant species (30.4%) collected in 2019.

The highest mean number of midges collected in nest boxes was in NRP (2.9 ± 1.4 [mean ± SE] insects per sampling) with maximum values being 48 biting midges per sampling in a nest box. The mean number of midges in BSR was much smaller and varied between 0.9 ± 0.2 (2012), 0.4 ± 0.2 (2018) and 0.1 ± 0.03 (2019) insects per sampling, with maximum values being 9 (2012), 24 (2018) and 4 females (2019). The highest abundance of *Culicoides* females in nest boxes was determined at the end of May (2018) and in the beginning of June (2012, 2019).

The difference between NRP and BSR catches was significant (*z* = 2.4, *N* = 11, 62, *p* = 0.02), but no differences between collection years were found in BSR (years 2012 *vs* 2018, *z* = 1.05, *N* = 14, 28, *p* = 0.29; 2018 *vs* 2019, *z* = 0.37, *N* = 28, 20, *p* = 0.71; 2012 *vs* 2019, *z* = 1.34, *N* = 14, 20, *p* = 0.18).

PCR-based analysis revealed that in total 13 (4.4%) *Culicoides* were infected with haemosporidian parasites. The sequencing revealed the presence of *Haemoproteus* and *Plasmodium* parasites in *Culicoides* females. Five genetic lineages of *Haemoproteus* parasites were detected in biting midges belonging to four *Culicoides* species (Table 2): *C. kibunensis* (1 female); *C. pictipennis* (5 females); *C. segnis* (3 females); *C. punctatus* (1 female). No biting midges infected by hemosporidians were collected in 2019. Two *C. kibunensis* and one *C. reconditus* contained DNA of *Plasmodium* parasites (Table 2).

Microscopic examination of all ten preparations of salivary glands extracted from *Haemoproteus*-positive *Culicoides* females did not reveal the presence of sporozoites.

## Discussion

The key result of our study is the detection of *H. minutus* sporozoites in the salivary glands of two *C. kibunensis* females. We also identified five *Culicoides* species as PCR positive for haemoproteid DNA, which may represent possible vectors of *Haemoproteus* parasites in our study sites. Results obtained from bird nest boxes revealed 11 *Culicoides* species being attracted by nesting birds. We have never obtained any *Culicoides* from empty nest boxes, for this reason, so we assume that insects were attracted by the presence of the birds.

It is known that sporogony of different *Haemoproteus* species with recorded sporozoite stages in salivary glands can be completed in four European *Culicoides* species: *C. impunctatus*, *C. nubeculosus*, *C. kibunensis* and *C. sphagnumensis* [2, 3, 7, 8, 12]. *Culicoides impunctatus* is one of the most abundant *Culicoides* species in North Europe as well as in our study sites [10, 11]. This species is a vector of 12 species of *Haemoproteus* parasites, as was proved experimentaly [12]. Midges of this *Culicoides* species are abundant in June in some localities, and this allows using them in experimental research [2, 12]. *Culicoides impunctatus* used to be considered a mammalophagic species [37], but cases of ornithophagic behavior of these biting midges have been documented [12]. Our study confirms ornithophagic *C. impunctatus* behavior as this insect was found visiting nest boxes of breeding birds and to be infected with avian haemoproteids at our study sites (Tables 1, 2).

*Culicoides nubeculosus* is the only Palearctic *Culicoides* species cultivated in the laboratory, which has permitted experimental studies on the sporogony of several *Haemoproteus* spp. [7, 13]. Some studies followed sporogonic development of haemoproteids till the sporozoite stage in wild *C. sphagnumensis* as well [3]. *Culicoides kibunensis* was detected as a vector of *Haemoproteus pallidus* (lineage hPFC1) in Lithuania, because two wild-caught individual midges of this species were detected harboring DNA as well as sporozoites of the parasite [8]. The results of our study showed that *C. kibunensis* is also a vector of *Haemoproteus minutus* because the DNA of two genetic lineages (hTURDUS2 and hTUPHI01) of this *Haemoproteus* parasite were detected in thoraxes of two females and sporozoites were present in salivary glands of the same insects. *Haemoproteus minutus* parasites are widespread in Common blackbirds *Turdus merula* in Europe and in our study sites [38]. This parasite is known to cause lethal disease in captive parrots in Europe [39]. Previous studies have shown that *H. minutus* (hTURDUS2) can be transmitted by wild-caught *C. impunctatus* [28] and laboratory-cultivated *C. nubeculosus* biting midges [7]. We have added *C. kibunensis* to the list of *H. minutus* vectors showing that this parasite seems to have low specificity to different *Culicoides* vectors. These three *Culicoides* species are not closely related, belonging to different *Culicoides* subgenera [40].

PCR-based testing of wild-caught insects for the presence of *Haemoproteus* DNA can be helpful to determine potential vectors of avian haemoproteids, but this method alone is insufficient to demonstrate that the insect is indeed a vector of the parasite [20]. Experimental studies indicate that avian malaria parasites can persist in non-competent insects for several weeks after initial blood meals because of the survival of ookinetes [21]. These parasites can be gained during blood meals on infected birds, and detection of parasite’s DNA proves information about the preferences for vertebrate hosts of biting midges in that particular site [21]. This information can also be helpful in detecting potential vectors, which later should be experimentally proven for vector competence. According to PCR-based testing, 11 *Culicoides* species are known to harbor *Haemoproteus* parasite DNA in Europe. These are *Culicoides alazanicus* [42], *Culicoides circumscriptus* [42], *C. festivipennis* [8, 16, 41], *C. impunctatus* [8, 20], *C. kibunensis* [8, 17, 18], *C. obsoletus* [8], *C. pictipennis* [8, 18, 41], *C. punctatus* [8, 20], *C. segnis* [17], *C. scoticus* [8, 18] and *C. paolae* [42]. We have detected avian haemosporidian parasites in biting midges belonging to five of these species (*C. impunctatus*, *C. kibunensis*, *C. segnis*, *C. pictipennis*, *C. punctatus*), and have now added *C. reconditus* to this list (Table 2) even thought *Plasmodium*, not *Haemoproteus,* DNA was detected in the *C. reconditus* female. The presence of *Plasmodium* DNA in *C. reconditus* as well as in *C. kibunensis* suggests that females of both species feed on birds [20]. However, *Culicoides* do not transmit avian *Plasmodium* parasites*,* though these can be gained during a blood meal from an infected bird and can be an illustration of abortive haemosporidian development in non-susceptible hosts [21].

We detected DNA of different *Haemoproteus* parasites in 5.2% of parous midges collected using a UV trap and 4.4% of midges collected from nest boxes (Tables 1, 2). *Haemoproteus tartakovskyi* (hSISKIN1) DNA was detected in *C. impunctatus*, *C. kibunensis* and *C. segnis* during this investigation*.* DNA of this parasite has been previously detected in *C. impunctatus*, *C. scoticus*, *C. obsoletus* and *C. kibunensis* [8, 20]. The sporozoites of *H. tartakovskyi* have been detected in salivary glands of experimentally infected *C. impunctatus* and *C. nubeculosus* [43, 44]. *Haemoproteus minutus* (hTURDUS2, hTUPHI01) was detected in four *Culicoides* species [*C. kibunensis*, *C. pictipennis*, *C. segnis* and *C. punctatus* (Tables 1, 2)]*.* DNA of *H. minutus* has already been detected in 11 *Culicoides* species [8, 16, 17, 20, 41, 42]. After successful experimental infections, *C. impunctatus* and *C. nubeculosus* were assigned as possible vectors of this parasite [7, 28]. Published data indicate broad susceptibility of the *C. impunctatus* and *C. nubeculosus* biting midges to many *Haemoproteus* parasites [7, 12] and in general show low vector specificity of the haemoproteids. However, *C. nubeculosus* has not been detected at our study site, and the distribution of *C. impunctatus* is very sporadic; therefore, other *Culicoides* species seem to be involved in the transmission of *H. minutus* and other *Haemoproteus* parasites.

*Haemoproteus majoris* (hCWT4), *Haemoproteus minutus* (hTURDUS2) and *Haemoproteus tartakovskyi* (hSISKIN1) were detected in three *C. segnis* females (Table 2). Three more genetic lineages of *Haemoproteus* (hSYAT01, hTURDUS3, hCOLL2) were detected in the same *Culicoides* species using a UV trap (Table 1). Four *Haemoproteus* genetic lineages (hCUKI1, hTUPHI01, hCCF4 and hROFI1) were detected in *C. segnis* in Europe by Synek et al. [17]. Based on these data, it is likely that *C. segnis* could be a potentially new vector of some haemoproteids. For confirmation of vector status, detailed experiments with confirmation of complete sporogony should be performed using this biting midge species in the future.

*Culicoides pictipennis* females were found in nest boxes and were infected with *H. minutus* (hTUPHI01) (Table 2). This species is of great interest because it is known to be ornitophagic [18, 20, 41, 45], and it is one of the earliest *Culicoides* species in the spring [18] that can infect birds after arrival from overwintering places.

The possibility to take part in the transmission of parasites depends on the host preference of biting midges. Only ornithophagic species can be involved in the transmission of avian *Haemoproteus* parasites. The blood meal analysis of *Culicoides* biting midges helps to understand the interaction between the insect and the bird. During the nesting time, birds are easy targets for blood sucking insects [2], so the collection of insects from bird nest boxes can help both to determine ornithophagic insect species and to identify infected insects. Seven out of 11 *Culicoides* species collected in nest boxes are already known to take blood meals on birds: *C. impunctatus*, *C. kibunensis*, *C. obsoletus*, *C. pictipennis*, *C. reconditus*, *C. segnis* and *C. festivipennis* [12, 16–18, 22, 45, 46]. Previously assigned as mammalophagic *Culicoides subfascipennis* and *C. pallidicornis* were collected in nest boxes; thus, they likely were naturally attracted by birds in the wild [47].

*Culicoides obsoletus* and *C. punctatus* are among the most abundant biting midges in North Europe [48, 49]. Thus, they should be considered for experimental research as potential vector candidates for *Haemoproteus* transmission. *Culicoides kibunensis*, *C. segnis* and *C. pictipennis* being the dominant species attacking birds, as determined in this study, were not known to be abundant at study sites on the Curonian spit. It was documented that *C. impunctatus* was the most dominant species in the Curonian spit and formed 82.1–99.7% of all *Culicoides* [11, 49, 50], and this species is still dominant, but only in some localities of the Curonian spit and only in June. Collection of biting midges from nest boxes showed that some dominant *Culicoides* species were not detected at all using other collection methods (light, netting, collection from humans) on the Curonian spit during earlier investigations [11, 50]. Probably the methodology that was applied for insect collection had a crucial impact on the species composition and number of collected insects. The method to collect biting midges from nest boxes may be of great importance not only with the target to find ornithophagic species [45] and potential vectors of avian haemoproteids, but also for biodiversity research and studies on changes of species composition and abundance of blood-sucking insects.

The diversity of *Culicoides* spp. in Europe is high [26], but available information about their involvement in transmission of *Haemoproteus* parasites is limited to a few *Culicoides* species. Therefore, using two collection methods, we found *Culicoides* species possibly involved in the transmission of haemoproteids and revealed which *Culicoides* species willingly take blood meals from birds. Information about *Culicoides* host preference and possible *Haemoproteus* vectors supplemented the missing information on the transmission of haemoproteids and will help to plan more detailed experimental studies on the sporogony process and untangle host-parasite interactions.

## Conclusions

Our results provide information about ornithophagic *Culicoides* species at the study sites. *Culicoides segnis*, *C. pictipennis* and *C. kibunensis*, being the dominant ornithophagic species and found to be infected with *Haemoproteus* parasites, should be considered as possible vectors of these parasites. *Culicoides kibunensis* is a new natural vector of *Haemoproteus minutus.* These data can help to initiate detailed experimental studies on sporogony of various *Haemoproteus* spp. parasites with the most abundant ornithophagic biting midge species. This study contributes to epizootiology of avian *Haemoproteus* infections by specifying *Culicoides* species that likely are responsible for the transmission of haemoproteids in Europe.

## Data Availability

The data that support findings of this study are included within the article.

## References

[1] Wirth W (1977). A review of the pathogens and parasites of the biting midges (Diptera: Ceratopogonidae). J Wash Acad Sci.

[2] Valkiūnas G (2005). Avian malaria parasites and other haemosporidia.

[3] Atkinson CT, Atkinson CT, Thomas NJ, Hunter BC (2008). *Haemoproteus*. Parasitic diseases of wild birds.

[4] Donovan TA, Schrenzel M, Tucker TA, Pessier AP, Stalis IH (2008). Hepatic hemorrhage, hemocoelom, and sudden death due to *Haemoproteus* infection in passerine birds: eleven cases. J Vet Diagn Invest.

[5] Ortiz-Catedral L, Brunton D, Stidworth MF, Elsheikha HM, Pennycott T, Schulze C, Braun M, Wink M, Gerlach H, Pendl H, Gruber AD, Ewen J, Perez-Tris J, Valkiūnas G, Olias P (2019). *Haemoproteus minutus* is highly virulent for Australasian and South American parrots. Parasites Vector.

[6] Borkent A. World species of biting midges (Diptera: Ceratopogonidae). New York: American Museum of Natural History. Accessed 2019-11-12.

[7] Bukauskaitė D, Iezhova TA, Ilgūnas M, Valkiūnas G (2019). High susceptibility of the laboratory-reared biting midges *Culicoides nubeculosus* to *Haemoproteus* infections, with review on *Culicoides* species that transmit avian haemoproteids. Parasitology.

[8] Bernotienė R, Žiegytė R, Vaitkutė G, Valkiūnas G (2019). Identification of a new vector species of avian haemoproteids, with a description of methodology for the determination of natural vectors of haemosporidian parasites. Parasites Vectors.

[9] Iezhova TA, Dodge M, Sehgal RN, Smith TB, Valkiunas G (2011). New avian *Haemoproteus* species (Haemosporida: Haemoproteidae) from African birds, with a critique of the use of host taxonomic information in hemoproteid classification. J Parasites.

[10] Carpenter S, Groschup MH, Garros C, Felippe-Bauer ML, Purse B (2013). *Culicoides* biting midges, arboviruses and public health in Europe. Antivir Res.

[11] Glukhova VM, Valkiūnas G (1993). On the fauna and ecology of biting midges (Ceratopogonidae: Culicoides) in the Curonian spit, the methods of their collection from the birds and experimental infection with haemoproteids (Haemosporidia: Haemoproteidae). Ekologija.

[12] Žiegytė R, Markovets MY, Bernotienė R, Mukhin A, Iezhova TA, Valkiūnas G, Palinauskas V (2017). The widespread biting midge *Culicoides impunctatus* (Ceratopogonidae) is susceptible to infection with numerous *Haemoproteus* (Haemoproteidae) species. Parasites Vector.

[13] Miltgen F, Landau I, Ratanaworabhan N, Yenbutra S (1981). *Parahaemoproteus desseri* n. sp.; Gametogonie et shizogonie chez I’hote naturel: *Psittacula roseate* de Thailande, et sporogonie experimentale chez *Culicoides nubeculosus*. Ann Parasitol Hum Comp.

[14] Bukauskaitė D, Chagas CRF, Bernotienė R, Žiegytė R, Ilgūnas M, Iezhova T, Valkiūnas G (2019). A new methodology for sporogony research of avian haemoproteids in laboratory-reared *Culicoides* spp., with a description of the complete sporogonic development of *Haemoproteus pastoris*. Parasites Vectors.

[15] Ferraguti M, Martinez-de la Puente J, Ruiz S, Soriguer R, Figuerola J (2013). On the study of the transmission network of blood parasites from SW Spain: diversity of avian haemosporidians in the biting midge *Culicoides circumscriptus* and wild birds. Parasites Vectors.

[16] Bobeva A, Ilieva M, Dimitrov D, Zehtindjiev P (2014). Degree of associations among vectors of the genus *Culicoides* (Diptera: Ceratopogonidae) and host bird species with respect to haemosporidian parasites in NE Bulgaria. Parasitol Res.

[17] Synek P, Munclinger P, Albrecht T, Votýpka J (2013). Avian haematophagous insects in the Czech Republic. Parasitol Res.

[18] Santiago-Alarcόn D, Havelka P, Pineda E, Segelbacher G, Schaefer HM (2013). Urban forests as hubs for novel zoonosis: blood meal analysis, seasonal variation in *Culicoides* (Diptera: Ceratopogonidae) vectors, and avian haemosporidians. Parasitology.

[19] Martínez-de la Puente J, Figuerola J, Soriguer R (2015). Fur or feather? Feeding preferences of species of *Culicoides* biting midges in Europe. Trends Parasitol.

[20] Bernotienė R, Valkiūnas G (2016). PCR detection of malaria parasites and related haemosporidians: the sensitive methodology in determining bird-biting insects. Malar J.

[21] Valkiūnas G, Kazlauskienė R, Bernotienė R, Palinauskas V, Iezhova TA (2013). Abortive long-lasting sporogony of two *Haemoproteus* species (Haemosporida, Haemoproteidae) in the mosquito *Ochlerotatus cantans*, with perspectives on haemosporidian vector research. Parasitol Res.

[22] Santiago-Alarcon D, Palinauskas V, Schaefer HM (2012). Diptera vectors of avian haemosporidian parasites: untangling parasite life cycles and their taxonomy. Biol Rev Camb Philos Soc.

[23] Tomas G, Merino S, Martinez-de la Puente J, Moreno J, Morales J, Lobato E (2008). A simple trapping method to estimate abundances ofblood-sucking flying insects in avian nests. Anim Behav.

[24] Gutsevich AV (1973). Blood-sucking midges of the genera *Culicoides* and Forcipomyia (Ceratopogonidae). Fauna USSR.

[25] Glukhova VM. Blood-sucking midges of the genera *Culicoides* and Forcipomyia (Ceratopogonidae). In: Fauna of the USSR. Dipteran insects. 1989;3.

[26] Mathieu B, Cêtre-Sossah C, Garros C, Chavernac D, Balenghien T, Carpenter S, Setier-Rio ML, Vignes-Lebbe R, Ung V, Candolfi E, Delécolle JC (2012). Development and validation of IIKC: an interactive identification key for *Culicoides* (Diptera: Ceratopogonidae) females from the Western Palaearctic region. Parasites Vectors.

[27] Dyce AL (1969). The recognition of nulliparous and parous *Culicoides* (Diptera: Ceratopogonidae) without dissection. Aust J Entomol.

[28] Žiegytė R, Palinauskas V, Bernotienė R, Iezhova TA, Valkiūnas G (2014). *Haemoproteus minutus* and *Haemoproteus belopolskyi* (Haemoproteidae): complete sporogony in the biting midge *Culicoides impunctatus* (Ceratopogonidae), with implications on epidemiology of Haemoproteosis. Exp Parasitol.

[29] Folmer O, Black M, Hoeh W, Lutz R, Vrijenhoek R (1994). DNA primers for amplification of mitochondrial cytochrome c oxidase subunit I from diverse metazoan invertebrates. Mol Mar Biol Biotechnol.

[30] Bensch S, Stjenman M, Hasselquist D, Ostman O, Hansson B, Westerdahl H, Pinheiro RT (2000). Host specificity in avian blood parasites: a study of *Plasmodium* and *Haemoproteus* mitochondrial DNA amplified from birds. Proc R Soc.

[31] Hellgren O, Waldenstrom J, Bensch S (2004). A new PCR assay for simultaneous studies of *Leucocytozoon*, *Plasmodium*, and *Haemoproteus* from avian blood. J Parasitol.

[32] Richardson DS, Jury FL, Blaakmeer K, Komdeur J, Burke T (2001). Parentage assignment and extra group paternity in a cooperative breeder: the Seychelles warbler (*Acrocephalus sechellensis*). Mol Ecol.

[33] Hellgren O, Bensch S, Malmqvist B (2008). Bird hosts, blood parasites and their vectors-associations uncovered by molecular analyses of blackfly blood meals. Mol Ecol.

[34] Valkiūnas G, Iezhova TA, Križanauskienė A, Palinauskas V, Bensch S (2008). A comparative analysis of microscopy and PCR-based detection methods for blood parasites. J Parasitol.

[35] Ciloglu A, Ellis VA, Bernotienė R, Valkiūnas G, Bensch S (2019). A new one-step multiplex PCR assay for simultaneous detection and identification of avian haemosporidian parasites. Parasitol Res.

[36] Hall TA (1999). A user-friendly biological sequence alignment editor and analysis program for Windows 98/98/NT. Nucleic Acid Symp Ser.

[37] Blackwell AA, Mordue J, Mordue W (1994). Identification of bloodmeals of the Scottish biting midge, *Culicoides impunctatus*, by indirect enzyme-linked immunosorbent assay (ELISA). Med Vet Entomol.

[38] Palinauskas V, Iezhova TA, Križanauskienė A, Markovets MY, Bensch S, Valkiūnas G (2013). Molecular characterization and distribution of *Haemoproteus minutus* (Haemosporida, Haemoproteidae): a pathogenic avian parasite. Parasitol Int.

[39] Olias P, Wegelin M, Zenker W, Freter S, Gruber AD, Klopfleisch R (2011). Avian malaria deaths in parrots. Eur Emerg Infect Dis.

[40] Borkent A, Dominiak P (2020). Catalog of the biting midges of the world (Diptera: Ceratopogonidae). Zootaxa.

[41] Bobeva A, Zehtindjiev P, Bensch S, Radrova J (2013). A survey of biting midges of the genus *Culicoides* Latreille, 1809 (Diptera: Ceratopogonidae) in NE Bulgaria, with respect to transmission of avian haemosporidians. Acta Parasitol.

[42] Veiga J, Martinez-de la Pueante J, Vaclav R, Figuerola J, Valera F (2018). *Culicoides paolae* and *C. circumscriptus* as potential vectors of avian haemosporidians in an arid ecosystem. Parasites Vectors.

[43] Valkiūnas G, Liutkevičius G, Iezhova TA (2002). Complete development of three species of *Haemoproteus* (Haemosporida, Haemoproteidae) in the biting midge *Culicoides* impunctatus (Diptera, Ceratopogonidae). J Parasitol.

[44] Žiegytė R, Bernotienė R, Palinauskas V, Valkiūnas G (2016). *Haemoproteus tartakovskyi* (Haemoproteidae): complete sporogony in *Culicoides nubeculosus* (Ceratopogonidae), with implications for avian haemoproteid experimental research. Exp Parasitol.

[45] Votypka J, Synek P, Svobodova M (2009). Endophagy of biting midges attacking cavity-nesting birds. Med Vet Entomol.

[46] Lassen SB, Nielsen SA, Kristensen M (2012). Identity and diversity of blood meal hosts of biting midges (Diptera: Ceratopogonidae: *Culicoides* Latreille) in Denmark. Parasites Vectors.

[47] Ayllón T, Nijhof AM, Weiher W, Bauer B, Allène X, Clausen PH (2014). Feeding behaviour of *Culicoides* spp. (Diptera: Ceratopogonidae) on cattle and sheep in northeast Germany. Parasites Vectors.

[48] Lassen SB, Nielsen SA, Skovgård H, Kristensen M (2011). Molecular identification of bloodmeals from biting midges (Diptera: Ceratopogonidae: *Culicoides* Latreille) in Denmark. Parasitol Res.

[49] Trukhan MN, Tereshkina NV, Liutkevičius G (2003). Peculiarities of the range of species and the ecology of midges (Diptera, Ceratopogonidae) on the Curonian spit. Vesci nacyanalnaj akademii navuk Belarusi.

[50] Liutkevičius G (2000). The new data on the epidemiology of bird haemoproteids (Haemosporida: Haemoproteidae) on the Curonian spit. Acta Zool Lithuan.

